# Impact of Insect Prey and Plant Food Sources on Development and Reproduction of the Phytozoophagous Mirid Bug, *Apolygus lucorum* (Meyer-Dür)

**DOI:** 10.3390/insects17050443

**Published:** 2026-04-22

**Authors:** Lili Wang, Lingyun Li, Baoyou Liu, Kongming Wu

**Affiliations:** 1 Yantai Academy of Agricultural Sciences, Yantai 265500, China; wsp0127@163.com (L.W.); lingyunmuzi@126.com (L.L.); baoyou1022@163.com (B.L.); 2 State Key Laboratory for Biology of Plant Diseases and Insect Pests, Institute of Plant Protection, Chinese Academy of Agricultural Sciences, Beijing 100193, China; 3 National Center of Technology Innovation for Comprehensive Utilization of Saline-Alkali Land, Dongying 257300, China

**Keywords:** omnivorous insect, life history, mixed diet, Miridae

## Abstract

*Apolygus lucorum* (Meyer-Dür) is a phytozoophagous crop pest. While the effects of plant-based diets on its development and reproduction have been extensively studied, the combined effects of plant- and prey-based diets on these traits remain poorly understood. This study systematically evaluated the effects of plant-only, prey-only, and mixed plant–prey diets on *A. lucorum* nymphal survival and development, as well as adult longevity and fecundity, under controlled laboratory conditions. The results demonstrate that diet composition significantly affected nymphal survival and developmental progression. Nymphs fed exclusively on prey (*Aphis gossypii* Glover or *Bemisia tabaci* (Gennadius) nymphs) failed to complete juvenile development. Although a diet of *Helicoverpa armigera* (Hübner) eggs alone enabled some individuals to reach adulthood, survival rates were significantly lower than those in mixed-diet treatments. Mixed feeding markedly improved nymphal survival, with the highest rates observed in groups fed green beans + *H. armigera* eggs and cotton leaves + *B. tabaci* nymph combinations (both 64.45%). The developmental duration was also influenced. Mixed diets, particularly green beans + *H. armigera* eggs, significantly shortened each instar and the total developmental time (11.04 ± 0.17 d), whereas a diet of cotton leaves alone prolonged development (19.45 ± 0.24 d). Adult longevity and reproductive output were likewise diet-dependent. The longest lifespans were recorded in adults fed green beans alone or green beans + *H. armigera* eggs, while the shortest lifespan was observed for those fed only cotton leaves. Successful oviposition was only achieved following four dietary treatments: green beans alone, green beans + *H. armigera* eggs, *H. armigera* eggs alone, and cotton leaves + *H. armigera* eggs. Among these, the green bean + *H. armigera* egg diet yielded the best reproductive performance, featuring the shortest pre-oviposition period (5.82 ± 0.60 d), the longest oviposition period (19.41 ± 1.68 d), and the highest mean fecundity per female (238.35 ± 25.51 eggs). This underscores the reproductive advantage of a mixed plant–prey diet. This study clarifies how dietary conditions shape the survival, development, and reproduction of *A. lucorum*, highlighting its strong reliance on nutritional quality for key life-history traits. These findings offer valuable insights into the ecological adaptations underlying the feeding behavior of this insect.

## Simple Summary

*Apolygus lucorum* (Meyer-Dür) is an agricultural pest that exhibits both herbivorous and carnivorous feeding behaviors. Previous studies have primarily focused on its herbivorous diet, but the combined effects of plant and prey food sources on its development and reproduction remain unclear. This study systematically evaluated the impacts of plant-only, prey-only, and mixed plant–prey diets on the growth, development, and reproduction of *A. lucorum* through controlled laboratory experiments. The results show that *A. lucorum* nymphs fed only with *Aphis gossypii* Glover or *Bemisia tabaci* (Gennadius) nymphs failed to develop into adults. Individuals consuming only cotton leaves completely developed but had short adult lifespans and were unable to reproduce. In contrast, mixed plant–prey diets significantly improved nymphal survival, shortened developmental duration, extended adult longevity, and enhanced fecundity. These findings highlight that a mixed plant–prey diet is essential for optimal development and reproduction in *A. lucorum*, providing valuable insights into its dietary adaptation mechanisms within agricultural ecosystems.

## 1. Introduction

*Apolygus lucorum* (Meyer-Dür) is a globally distributed omnivorous pest that inflicts significant economic damage on cotton, fruit trees, and vegetable crops across Asia, Europe, North America, and Northern Africa [1,2,3]. Its piercing–sucking feeding behavior causes direct plant tissue injury, leading to symptoms such as growth stunting, abscission of buds and bolls, and leaf malformation [4,5]. Beyond phytophagy, *A. lucorum* also preys on small arthropods, including aphids, whiteflies, and lepidopteran eggs [6,7]. This mixed plant–prey feeding habit—which involves the utilization of both plant and prey resources—represents a key adaptive strategy that enhances its nutritional intake and buffers against food scarcity, a trait common among many omnivorous insects [8,9].

Omnivorous insects can be categorized into two distinct trophic types based on the proportion of prey and plant food in their overall nutritional intake: phytozoophagous or zoophytophagous [10,11]. These two types exhibit fundamental differences in feeding strategies, mouthpart structures, and ecological niches. Zoophytophagous insects primarily rely on other small arthropods as their main nutritional source. Their mouthparts and behavioral traits are more adapted to predation. Typically, they do not actively damage plants. However, when prey is scarce in the environment or there is a shortage of prey food due to high population densities, they will feed on plants to sustain their survival and reproduction [12,13,14]. *Nesidiocoris tenuis* (Reuter), *Orius sauteri* (Poppius), and *Brontocoris tabidus* (Signoret) all fall into this category. These insects play a significant role in biological control, although their plant-feeding behavior can occasionally cause minor damage to crops [15,16,17,18]. In contrast, phytozoophagous insects primarily feed on plant tissues, which are essential for their survival. Their mouthparts and digestive systems are more suited to plant feeding, allowing them to complete their entire life cycle on a plant-based diet. However, these insects can significantly enhance their nutritional status by supplementing their diet with prey food, obtaining essential nitrogen sources and specific amino acids and thereby improving their growth, development, reproductive capacity, and population fitness [11,19]. Notable examples include *Lygus lineolaris* (Palisot de Beauvois) and *Lygus hesperus* Knight, two major agricultural pests in North America that primarily feed on plants but exhibit enhanced population growth through predation [20]. Similarly, *Adelphocoris lineolatus* (Goeze) and *Adelphocoris suturalis* (Jakovlev), common mirid bugs in Chinese cotton fields, also display phytozoophagous characteristics [21,22].

*Apolygus lucorum* is a typical phytozoophagous insect in terms of its nutritional strategy. Under natural conditions, it primarily depends on the tender tissues of host plants as its fundamental nutritional source and can complete the entire developmental cycle from egg to adult on a plant-based diet alone [23,24]. However, both cotton field observations and laboratory studies have confirmed that *A. lucorum* exhibits active predatory behavior, actively searching for and feeding on prey food sources such as *Aphis gossypii* Glover nymphs, *Bemisia tabaci* (Gennadius) nymphs, and *Helicoverpa armigera* (Hübner) eggs [6,7]. This mixed nutritional strategy, which is primarily plant-based but supplemented with prey food, may be the crucial ecological basis for its ability to adapt widely to various hosts, maintain a stable population, and occasionally cause outbreaks in agricultural ecosystems. It is worth noting that unlike zoophytophagous predatory insects, *A. lucorum* has a relatively low dependence on prey food, and its predatory behavior serves more as a supplement to plant nutrition rather than a substitute [11,25].

To date, research on *A. lucorum* has predominantly focused on population dynamics, damage mechanisms, and chemical control [26,27,28]. While some studies have addressed its nutritional ecology, these have primarily examined the effects of a single host plant on development and reproduction [29,30]. However, several key questions remain largely unexplored: the specific role of prey food in its life history, the optimal nutritional ratio of plant to prey food, and the impact of different food combinations on fitness. There is a particular lack of experimental evidence comparing the effects of purely plant-based, purely prey-based, and mixed diets on growth, development, and reproduction under strictly controlled conditions. Moreover, given the distinct stage-specific differences in insect nutritional strategies, inferring overall life history nutritional adaptations from the results of a single developmental stage may not provide a comprehensive understanding of its ecological strategy.

This study systematically evaluated the effects of prey-only, plant-only, and mixed plant–prey diets on the survival and development of *A. lucorum* nymphs, as well as on adult longevity and fecundity, through controlled laboratory experiments. The primary objectives were to elucidate the nutritional dependence of *A. lucorum* on plant and prey food sources and to understand the ecological adaptation mechanisms underlying its omnivorous strategy. It is important to clarify that *A. lucorum* is a phytozoophagous pest whose primary nutritional source is plants, and its population outbreaks are driven mainly by feeding damage to host plants. Therefore, the findings of this study are not intended to propose direct “nutritional management” control tactics. Instead, the core applied value of these results lies in providing theoretical parameters for improving population forecasting and risk assessment. By clarifying the effects of different food combinations on the life-history parameters of *A. lucorum*, this study helps to understand how the spatiotemporal synchrony of host plants and prey resources in the field influences pest population dynamics, thereby providing a basis for developing more accurate monitoring and early warning models.

## 2. Materials and Methods

### 2.1. Insect Rearing

A laboratory colony of *A. lucorum* was established from adults originally collected in Langfang City, Hebei Province, China (39°30′ N, 116°36′ E). The insects were reared continuously under controlled conditions [31]. Nymphs were maintained in transparent plastic containers (19.5 cm × 13.4 cm × 7.2 cm) with fresh or split green bean (*Phaseolus vulgaris* L.) pods, at a 200–300 individuals per container. Several folded filter paper strips (1 cm × 20 cm) were placed at the bottom of each container to facilitate insect movement. The container opening was covered with medical gauze and secured with the lid to prevent escape, and a square opening (7 cm × 7 cm) was cut in the lid for ventilation. Upon reaching adulthood, adults were also fed with green beans, and the ends of the beans were cut at a slant to serve as an oviposition substrate. A cotton ball soaked with 10% honey–water solution was placed on the gauze cover to provide supplement nutrition. After oviposition, green beans bearing egg masses were placed in a ventilated area for 4–5 days and then transferred to clean containers. Egg hatching was monitored daily. Newly hatched nymphs from the same day were transferred to another clean container with fresh green bean pods and reared as described above for subsequent generations. Rearing conditions were maintained at 26 ± 1 °C, 70 ± 5% (RH), and a photoperiod of 14 L/10 D.

The *H. armigera* colony was initiated from individuals collected in Xinxiang, Henan Province (35°18′ N, 113°54′ E), and reared on an artificial diet following the formula described by Liang et al. [32]. Adults were provided with 10% sugar solution as a nutritional supplement. The rearing conditions were 26 ± 1 °C, 70 ± 5% (RH) and a photoperiod of 16 L/8 D.

The experimental populations of *A. gossypii* and *B. tabaci* were collected from cotton (the same variety as described in Section 2.2.1) fields in Langfang and were used directly in experiments without laboratory rearing.

### 2.2. Experimental Methods

#### 2.2.1. Plant Materials

Green bean (*Phaseolus vulgaris* L.) pods were purchased fresh from a local farmers’ market, representing a conventional locally cultivated variety; the specific variety was not identified.

Cotton leaves were collected from the conventional cotton variety Shiyuan 321 (also known as Jimian 24, *Gossypium hirsutum* L.), which was a widely cultivated variety in the Yellow River cotton region of China. Cotton plants were sown in May 2010 in the experimental field of the Langfang Pilot Base of the Chinese Academy of Agricultural Sciences (Langfang, Hebei, China) and managed under conventional cultivation practices with standard fertilization and irrigation. From June to August 2010 (plant age approximately 40–120 days), fully expanded leaves were collected from the middle to upper parts of the plants. Whole leaves were used in the experiments, and all leaves were used fresh on the day of collection.

#### 2.2.2. Effects of Different Diets on *A. lucorum* Development

(a) Diet treatments and experimental conditions: Nine diet treatments were established, including *H. armigera* eggs, *A. gossypii* nymphs, *B. tabaci* nymphs, green beans, cotton leaves, green beans + *H. armigera* eggs, cotton leaves + *H. armigera* eggs, cotton leaves + *A. gossypii* nymphs, and cotton leaves + *B. tabaci* nymphs. Each piece of gauze or leaf was ensured to contain no less than 50 *H. armigera* eggs, *A. gossypii* nymphs, or *B. tabaci* nymphs to meet the feeding requirements of *A. lucorum*. All experiments were conducted in a controlled environmental chamber with conditions set at 26 ± 1 °C, 70 ± 5% (RH), and a photoperiod of 14 L/10 D.
(b) Nymph rearing and observation: Each diet type was placed in a flat-bottomed glass test tube (diameter 2.5 cm, height 7.5 cm). A single, newly hatched (within 24 h of hatching) *A. lucorum* nymph was then introduced into each tube. The tube opening was covered with 80-mesh nylon gauze secured with a rubber band to prevent escape. Nymphal survival and developmental progress were recorded daily, and fresh food was provided. Observations continued until all individuals either died or emerged as an adult. Each treatment consisted of 30 nymphs, replicated three times.
(c) Adult pairing and oviposition observation: Upon adult emergence, virgin males and females (≤24 h post-emergence) were paired. Each pair was confined in a vial containing the corresponding diet. The tube openings were similarly sealed with 80-mesh nylon netting, with a piece of filter paper placed on the net for the female to lay eggs, and a moistened cotton ball added on top for water supply and humidity maintenance. The survival of adults was recorded daily; fresh food and filter paper was provided. The replaced green beans, filter papers, and cotton leaves were collected and examined under a microscope to count the number of eggs laid. Twenty pairs of adults were used for each treatment, with three replicates.
(d) Egg hatching observations: Green beans, filter papers, and cotton leaves with *A. lucorum* eggs were collected daily and placed in tubes with moistened cotton balls for humidity maintenance. Egg hatching was checked daily starting from the third day; the number of hatched eggs was recorded, and newly hatched nymphs were promptly removed.

### 2.3. Data Analysis

All statistical analyses were performed using SAS 9.4 (SAS Institute Inc., Cary, NC, USA). All data are presented as mean ± standard error (SE) unless otherwise specified.

Prior to analysis, the normality of the data was assessed using the Shapiro–Wilk test, and the homogeneity of variances was assessed using Levene’s test for all variables, including nymphal survival rate, nymphal developmental duration, adult pre-oviposition period, oviposition period and fecundity. For variables that did not meet the assumptions of normality and homoscedasticity, appropriate data transformations were applied. Specifically, nymphal survival rate (percentage data) was arcsine square-root transformed before analysis to improve normality. Other variables that met the assumptions were analyzed without transformation.

The effects of diet treatment on nymphal developmental duration (both individual instars and total), adult pre-oviposition period, oviposition period, and female fecundity were analyzed using one-way analysis of variance (ANOVA). When significant differences were detected (*p* < 0.05), means were separated using Tukey’s Honestly Significant Difference (HSD) test for multiple comparisons.

Adult longevity was analyzed using the Kaplan–Meier method with the SAS PROC LIFETEST procedure. Survival curves were compared using the log-rank (Mantel–Cox) test. Data are presented as median survival time (days) with 95% confidence intervals.

## 3. Results

### 3.1. Effects of Different Diets on Nymphal Survival Rate

**Figure 1:**
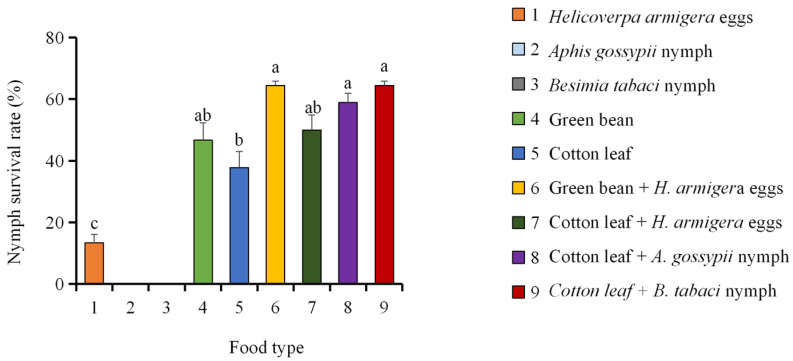
Nymphal survival rate of *Apolygus lucorum* fed on different diets. Different lowercase letters indicate significant differences among diet treatments (one-way ANOVA with Tukey’s HSD post hoc test, *p* < 0.05). Survival rate data were arcsine square-root transformed prior to analysis to meet normality assumptions. Data are presented as mean ± SE.

The survival rate of *A. lucorum* nymphs was significantly affected by different diet treatments. After prey-only diet treatments, nymphal development was severely impaired, while in treatment groups fed exclusively on *A. gossypii* nymphs or *B. tabaci* nymphs, all individuals died during the nymphal stage and failed to reach adulthood. In the group fed exclusively on *H. armigera eggs*, although a small proportion of individuals successfully developed into adults, the nymphal survival rate remained significantly lower than that of other treatments. In contrast, mixed diets (plant–prey) substantially improved nymphal survival rate. Among these, the combinations of green beans + *H. armigera* eggs and cotton leaves + *B. tabaci* nymphs resulted in the highest nymphal survival rates, both at 64.45%. The nymphal survival rates of the remaining treatment groups descended in the following order: cotton leaves + *A. gossypii* nymphs > cotton leaves + *H. armigera* eggs > green beans > cotton leaves (Figure 1). These findings indicate that a mixed nutritional mode combining plant and prey food sources is essential for nymphal survival, whereas a prey-only diet is insufficient to support complete development.

### 3.2. Effects of Different Diets on Nymphal Developmental Duration

The developmental duration of each instar and the total nymphal period of *A. lucorum* varied significantly among different diet treatments (Table 1). Statistical analysis revealed significant effects of diet treatment on each instar and the total nymphal period (1st instar: *F* = 40.42, *df* = 6,12, *p* < 0.01; 2nd instar: *F* = 41.032, *df* = 6,12, *p* < 0.05; 3rd instar: *F* = 17.79, *df* = 6,12, *p* < 0.05; 4th instar: *F* = 13.18, *df* = 6,12, *p* < 0.05; 5th instar: *F* = 5.54, *df* = 6,12, *p* < 0.05; total nymphal period: *F* = 72.88, *df* = 6,12, *p* < 0.05). Among the mixed diet treatments, the addition of *H. armigera* eggs significantly shortened the developmental duration of each nymphal instar. Individuals fed green beans + *H. armigera* eggs exhibited the shortest developmental durations for the first, third, and fourth instars, while those fed cotton leaves + *H. armigera* eggs showed the shortest durations for the second and fifth instars.

Regarding single-food treatments, nymphs fed exclusively on *A. gossypii* or *B. tabaci* nymphs failed to survive. Nymphs fed exclusively on *H. armigera* eggs were able to completely develop, albeit with low survival rates (Figure 1), and their total nymphal developmental duration was like that of nymphs fed on green beans alone. Nymphs fed exclusively on cotton leaves exhibited the longest total nymphal developmental duration. Among the mixed diet treatments, the green bean + *H. armigera egg* treatment resulted in the shortest total nymphal developmental duration, followed by cotton leaves + *H. armigera* eggs, cotton leaves + *B. tabaci* nymphs, and cotton leaves + *A. gossypii* nymphs. Supplementing plant-based diets with insect prey (e.g., *H. armigera* eggs, *A. gossypii* nymphs, or *B. tabaci* nymphs) significantly shortened the total nymphal developmental duration of *A. lucorum* (Table 1).

### 3.3. Effects of Different Diets on Adult Longevity and Reproduction

**Figure 2:**
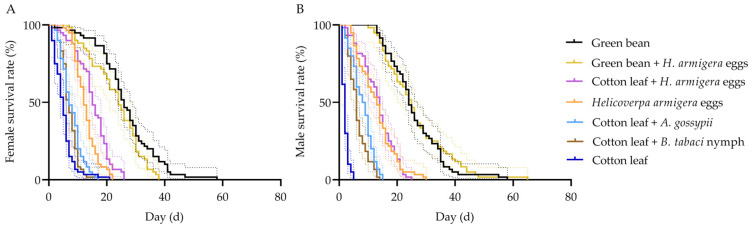
Kaplan–Meier survival curves of adult *Apolygus lucorum* fed on different diets. (**A**) Female adults; (**B**) Male adults. Survival curves were compared using the log-rank (Mantel–Cox) test. Different colors represent different diet treatments. Data are presented as survival probability over time (days).

The daily survival rates of female and male *A. lucorum* adults under different diet treatments are shown in Figure 2. Because no individuals survived to the adult stage when fed exclusively on *A. gossypii* or *B. tabaci* nymphs, these two treatments were excluded from the adult longevity analysis.

Among all treatments, adults fed green beans + *H. armigera* eggs and green beans alone exhibited the highest survival rates. On day 20, the survival rates of females and males were 67% and 68.33% for the green beans + *H. armigera* egg treatment, and 82% and 73.33% for the green bean alone treatment, respectively. Adults fed *H. armigera* eggs alone and cotton leaves + *H. armigera* eggs showed intermediate survival rates. On day 20, the survival rates of females and males were 8.33% and 16.67% for the *H. armigera* eggs alone treatment, and 23.33% and 16.67% for the cotton leave + *H. armigera* egg treatment, respectively. In contrast, survival declined most rapidly in the cotton leaves alone, cotton leaves + *A. gossypii* nymphs, and cotton leaves + *B. tabaci* nymph treatments. On day 20, except for a female survival rate of 1.67% in the cotton leaves alone treatment, all other survival rates in these three treatments were 0%.

Significant differences were found in the pre-oviposition period, oviposition period, and fecundity of female *A*. *lucorum* under different diet treatments (Table 2). Among the four treatments where oviposition occurred, females fed green beans + *H. armigera* eggs had the shortest pre-oviposition period, followed by those fed *H. armigera* eggs alone, while females fed cotton leaves + *H. armigera* eggs required the longest pre-oviposition period (*p* < 0.05). The oviposition period was longest for females fed green beans alone, followed by those fed green beans + *H. armigera* eggs; both were significantly longer than those fed *H. armigera* eggs alone or cotton leaves + *H. armigera* eggs (*p* < 0.05). Fecundity was highest in females fed green beans + *H. armigera* eggs, which was significantly higher than all other treatments (*p* < 0.05), followed by those fed green beans alone.

Taken together, these findings indicate that diet composition not only affects the survival of female *A. lucorum* but also significantly regulates their reproductive duration and fecundity. The green beans + *H. armigera* eggs group consistently outperformed all others regarding time to sexual maturation, the length of the oviposition period, and fecundity. This synergistic combination of plant and prey nutrients appears to best match the reproductive nutritional requirements of *A. lucorum*.

## 4. Discussion

Our finding that *A*. *lucorum* benefits significantly from a mixed plant–prey diet to optimize life history performance aligns with the nutritional ecology generally observed in omnivorous mirids. Accumulating evidence indicates that a mixed feeding mode is critical for development and population maintenance in Miridae [33,34]. For instance, *N*. *tenuis* nymphs fail to develop when fed exclusively on plant tissues (e.g., buckwheat (*Fagopyrum esculentum* Moench), alyssum (*Lobularia maritima* (L.) Desv.), or cotton (*Gossypium hirsutum* L.)), but when supplemented with *B. tabaci* prey, they not only complete development successfully but also exhibit a significantly shortened nymphal period [35]. Similarly, *Macrolophus pygmaeus* (Rambur) exhibits elevated mortality and prolonged development under plant-only conditions [36,37]. Interspecific variation in dietary dependence is also evident: *Lygus pratensis* (Linnaeus) exhibits stronger plant-feeding bias (preferring plants such as oilseed rape (*Brassica napus* L.), oak-leaved goosefoot (*Chenopodium glaucum* L.), alfalfa (*Medicago sativa* L.), and cotton) akin to *A. lucorum* [38,39], whereas *N. tenuis* shows a greater requirement for insect prey (e.g., *B. tabaci*, *Frankliniella occidentalis* (Pergande), *Spodoptera litura* (Fabricius) larvae, and *Tetranychus urticae* Koch) [40,41]. Collectively, these studies demonstrate that the synergistic effects of plant and prey nutrients not only provide comprehensive nutritional support for mirid bugs but also enhance their ecological adaptability in heterogeneous environments. The evolution of this nutritional strategy may be the key foundation for this group to become major pests or biological control agents in agricultural ecosystems.

A particularly noteworthy finding is that *A. lucorum* females fed exclusively on green beans achieved substantial fecundity (105.50 eggs/female), whereas those fed exclusively on cotton leaves laid no eggs at all, despite both being leguminous and malvaceous plants, respectively. This disparity suggests that plant nutritional quality, rather than the mere presence of plant material, determines reproductive success. Green beans are rich in proteins, sugars, and moisture, which may provide the necessary energy and building blocks for vitellogenesis [42,43]. In contrast, cotton leaves contain higher levels of secondary metabolites such as gossypol and tannins, which are known to deter herbivory and may impair reproductive physiology [44]. Similar findings have been reported for *L*. *hesperus*, which showed reduced fecundity on cotton compared to bean plants [45].

The fecundity of *A. lucorum* was significantly higher when green beans were supplemented with *H. armigera* eggs than when green beans were provided alone, highlighting the nutritional complementarity between plant and animal food sources. From a nutritional metabolic perspective, a prey-based diet (e.g., *H. armigera* eggs or *A. gossypii* nymphs) serves as a critical source of proteins, lipids, and essential amino acids, which directly contribute to development, vitellogenesis, and energy metabolism [46,47]. Notably, in the treatment consisting exclusively of *H. armigera* eggs, some individuals successfully completed nymphal development and achieved limited reproduction, whereas nymphs fed exclusively on *A. gossypii* or *B. tabaci* nymphs failed to reach the adult stage. This discrepancy may reflect differences in nutritional composition among prey types: lepidopteran eggs may contain more balanced proportions of proteins, lipids, and essential amino acids, thereby supporting complete nymphal development. By contrast, a plant-based diet (e.g., green beans or cotton leaves) provides carbohydrates, water, and certain vitamins that support basal metabolism and water regulation [42,43]. Furthermore, gut endosymbionts may play a crucial role in nutrient assimilation, enabling *A. lucorum* to efficiently utilize diverse food sources [48,49]. Under mixed dietary conditions, the synergistic action of plant and prey nutrients optimizes nutritional ratios and improves nutrient utilization efficiency, allowing individuals to better adapt to the metabolic demands of different developmental stages.

Several limitations of this study should be acknowledged. First, all experiments were conducted under controlled laboratory conditions, which do not fully capture the spatiotemporal heterogeneity of food resources, climatic fluctuations, or interspecific competition inherent to field environments. Therefore, extrapolation of these findings to natural populations warrants cautious validation under more ecologically realistic settings. Second, although representative diet combinations were examined, a continuous gradient of plant–prey nutritional ratios were not systematically established, which limits our capacity to quantitatively disentangle the relative contributions of the two food types. Third, our assessment focused on individual life-history traits; population-level dynamics, behavioral responses, and physiological stress tolerance remain to be investigated. Future research should integrate field cage experiments, dose–response models, and metabolomic approaches to further elucidate the nutritional requirements of *A. lucorum*.

## 5. Conclusions

This study confirms that *Apolygus lucorum* employs a typical phytozoophagous feeding strategy, requiring both plant and prey nutrients to achieve optimal development and reproduction. Mixed plant–prey diets significantly improved nymphal survival, shortened developmental time, extended adult longevity, and maximized fecundity. Among all treatments, the combination of green beans and *Helicoverpa armigera* eggs consistently performed best across all measured parameters.

From an applied perspective, these findings do not support nutritional management as a stand-alone control tactic for *A. lucorum*, given its primary dependence on plant feeding. Nevertheless, they offer valuable insights for population monitoring and outbreak early warning. Specifically, field risk assessment should account for the spatial and temporal co-occurrence of high-quality host plants and suitable prey resources, as such conditions may elevate outbreak risks. This study therefore provides a theoretical basis for optimizing field sampling protocols and developing multi-factor predictive models for *A. lucorum* management. Future research should validate these laboratory findings under semi-field or field conditions to quantify how the co-occurrence of food resources influences population dynamics.

## Figures and Tables

**Table 1:** Effects of different diets on the nymphal development duration of *Apolygus lucorum*.

Diet Treatment	Nymphal Development Duration (d)					
	1st Instar	2nd Instar	3rd Instar	4th Instar	5th Instar	Total
*Helicoverpa armigera* eggs	1.83 ± 0.03 c	4.17 ± 0.08 b	2.08 ± 0.08 b	2.58 ± 0.15 bc	2.08 ± 0.06 ab	12.75 ± 0.02 c
*Aphis gossypii* nymphs	—	—	—	—	—	—
*Bemisia tabaci* nymphs	—	—	—	—	—	—
Green beans	1.89 ± 0.16 c	4.04 ± 0.22 b	2.37 ± 0.10 b	2.37 ± 0.19 bc	1.97 ± 0.06 ab	12.64 ± 0.43 cd
Cotton leaves	2.02 ± 0.05 bc	6.83 ± 0.28 a	3.86 ± 0.21 a	4.71 ± 0.18 a	2.03 ± 0.06 ab	19.45 ± 0.24 a
Green beans + *H. armigera* eggs	1.09 ± 0.07 d	4.00 ± 0.14 b	2.00 ± 0.01 b	1.94 ± 0.22 c	2.02 ± 0.05 ab	11.04 ± 0.17 d
Cotton leaves + *H. armigera* eggs	2.95 ± 0.16 a	2.39 ± 0.19 c	2.37 ± 0.23 b	2.53 ± 0.07 bc	1.76 ± 0.04 b	12.01 ± 0.19 cd
Cotton leaves + *A. gossypii* nymphs	2.89 ± 0.07 a	4.15 ± 0.09 b	3.45 ± 0.19 b	3.22 ± 0.35 b	2.32 ± 0.20 a	16.04 ± 0.49 b
Cotton leaves + *B. tabaci* nymphs	2.52 ± 0.09 ab	3.59 ± 0.18 b	2.37 ± 0.13 b	2.60 ± 0.22 bc	2.15 ± 0.13 ab	13.23 ± 0.32 c

Data are presented as mean ± SE. Different lowercase letters in the same column indicate significant differences among treatments (one-way ANOVA, Tukey’s HSD test, *p* < 0.05). “—” indicates that nymphal development could not be completed or was not observed. *n* = 30 per treatment at the start of each instar.

**Table 2:** Effects of different diets on the reproductive performance of female *Apolygus lucorum*.

Diet Treatment	Pre-Oviposition Period (d)	Oviposition Period (d)	Fecundity (Eggs per Female)
*Helicoverpa armigera* eggs	6.54 ± 0.64 b	6.46 ± 0.94 b	39.54 ± 10.65 b
*Aphis gossypii* nymphs	—	—	0
*Bemisia tabaci* nymphs	—	—	0
Green beans	7.19 ± 0.62 b	20.50 ± 3.58 a	105.50 ± 19.63 b
Cotton leaves	—	—	0
Green beans + *H. armigera* eggs	5.82 ± 0.60 b	19.41 ± 1.68 a	238.35 ± 25.51 a
Cotton leaves + *H. armigera* eggs	10.46 ± 0.37 a	5.23 ± 0.78 b	34.15 ± 5.20 b
Cotton leaves + *A. gossypii* nymphs	—	—	0
Cotton leaves + *B. tabaci* nymphs	—	—	0

Data are presented as mean ± SE. Different lowercase letters within the same column indicate significant differences (one-way ANOVA, Tukey’s HSD test, *p* < 0.05). “—” indicates that females died before reaching the oviposition stage under the corresponding diet treatment, and thus fecundity was zero.

## Data Availability

The original contributions presented in this study are included in the article. Further inquiries can be directed to the corresponding author.
